# ABRF 2026 Meeting Report

**DOI:** 10.7171/001c.162770

**Published:** 2026-06-08

**Authors:** Joshua Z. Rappoport, Marie Adams

**Affiliations:** 1 Research Infrastructure & Operations Boston College; 2 Genomics Core Van Andel Institute

**Keywords:** ABRF, Conference

## Abstract

The 2026 Association of Biomolecular Resource Facilities (ABRF) annual meeting took place in Pittsburgh, Pennsylvania, from March 28–31, 2026, and brought scientific core directors, core staff, institutional leadership, and industry partners together into community to discuss their challenges, goals, and successes in supporting and advancing Shared Research Resources (SRRs). The tagline for the meeting was, “Innovating at the intersection of science, technology, and collaboration.” For many in the SRR community, the ABRF conference is a highlight of the year, and for the over 800 attendees, the meeting this year in Pittsburgh did not disappoint!

The 2026 Association of Biomolecular Resource Facilities (ABRF) annual meeting took place in Pittsburgh, Pennsylvania from March 28–31, 2026 and brought scientific core directors, core staff, institutional leadership, and industry partners together into a community to discuss their challenges, goals, and successes in supporting and advancing shared research resources (SRRs). The tagline for the meeting was, “Innovating at the intersection of science, technology, and collaboration.” For many in the SRR community, the ABRF conference is a highlight of the year, and for the over 800 attendees, the meeting this year in Pittsburgh did not disappoint!

The conference began as usual with premeeting workshops, including the 10th anniversary rendition of the Lean Management for SRR workshop, which is always a crowd pleaser. A major conference theme was commitment to the technical staff that makes SRRs possible, and how we can bolster support for professional development and staff advancement at all levels. This theme was highlighted in the opening keynote address presented by Mark Kristiansen from University College London (UCL). Mark discussed the growth journey of UCL Genomics, which he leads, into a global powerhouse supporting biomedical research in London and throughout the world; the growth was largely a result of creating a culture that empowers his team. Mark focused on unique opportunities he has provided, with both an educational and a technical focus, to allow his staff to make tangible, independent research contributions to produce high-quality results for a wide array of customers and stakeholders and develop their skills and independent career paths.

The next morning, the conference day started with another fantastic presentation from another speaker from across the pond: Kelly Vere from the University of Nottingham. Building on the keynote address, Kelly Vere MBE brought Mark Kristiansen’s high-level, aspirational vision down to earth with her plenary discussion on the Technician Commitment, an international initiative she began nearly a decade ago with the aim of ensuring that technical staff receive the recognition, career progression tools, and resources necessary to navigate the complex hierarchies within scientific, medical, and technical fields.

Kelly spoke of her early roles as a technician and how those experiences focused her desire to promote the role that technical staff play in advancing research. The Technician Commitment provides tangible mechanisms to promote visibility, stability, career development, and recognition for skilled technical professionals. Crucially, the initiative also provides infrastructure and resources for workforce development through the UK Institute for Technical Skills and Strategy.

The Technician Commitment now has over 120 signatory and supporter organizations, and although it started in the UK, it is now spreading globally. Kelly’s presentation described the process of integrating the Technician Commitment into administrative workflows, and how she and her team brilliantly managed to get many of the most prestigious research and educational institutions in the UK to sign on to the initiative. Kelly’s work has attracted international attention and funding from Research England and UK Research and Innovation (UKRI) to establish the UK Institute for Technical Skills & Strategy, which is now the home of the Technician Commitment. In addition to serving as a fantastic case study for development of new initiatives to support professional development, the ABRF is currently exploring ways in which it can collaborate with Kelly, her team, and the UK Institute for Technical Skills & Strategy.

As Kelly’s work and presentation are applicable across all types of technical fields, the theme of cross-disciplinarity was further emphasized by the session entitled, “Out of the Silo and Into the Future: Core Facilities Across Disciplines.” Josh Stapleton, who runs the Materials Characterization Lab at the Penn State Materials Research Institute, described his large and dynamic SRR, highlighting support they are providing to researchers from physical sciences disciplines and beyond, including users from outside Penn State.

Also speaking in this session was ABRF past-president Thayumanasamy “Soma” Somasundaram from Florida State University, where he leads the X-Ray Crystallography Facility in the Institute of Molecular Biophysics. Soma described his experience building his own lab equipment as a PhD student and post-doc and his interests and experiences in both materials science and biomedical research. He also provided information on the intersection between teaching and training that he and his facility support through a three-week facility bootcamp for new PhD students with hands-on learning activities.

Artificial intelligence (AI) represents another topic relevant to all types of SRRs; the session entitled “How AI Can Be Used to Improve Core & Platform Operations” proved interesting and practically useful to those who attended. James Chambers from UMass Amherst described his experience creating a chatbot to support users of the microscopy and imaging SRR that he leads. In particular, his insights into training and guardrails will prove extremely helpful to the members of our community currently looking to develop similar tools.

In the same session, Melissa Mann from St. Jude Children’s Research Hospital gave a deep dive into how to write effective prompts for generative AI tools. Sean Schaffer from Vanderbilt described best practices in employing AI as a tool to improve efficiency and provided advice on how to adapt AI to streamline and enhance existing workflows. Overall, the session provided an excellent balance between case studies, tips and tricks, and an overall view of how AI is rapidly changing how we all work.

Kelly Vere MBE from the University of Nottingham was back again for the session, “Career Development for Staff: How to Make the Step From Staff to Leadership,” joined by Heidi Monroe from the University of Pittsburgh and Barbara Fransway from University of Arizona, each of whom provided compelling insights from their own personal experiences placed within the broader contexts of academic career development across different types of institutions. Although the similarities were striking across each of their stories, the individual perspectives and contexts provided excellent contrasts.

Kelly further elucidated about the Technician Commitment as a tool for career development through institutional means, and its impact on individuals from numerous different subject areas was extremely interesting. Other topics that were raised included an open and frank discussion of the extremely limited career ladder ceiling for technical staff in many roles across different types of institutions, which seemed to correlate with a lack of focus on professional development activities, research outputs, and institutional service. All in attendance took away something actionable regardless of background, role, and level of seniority.

Throughout many of the technical sessions, there was a focus on the growing interdisciplinary nature of SRR environments and the additional challenges this can create in communication, shared knowledge across practical domains, and how to best structure SRRs and teams to respond to greater unsiloing. Additionally, with funding changes, many labs have started to view SRR staff and resources as extensions of their own research programs. In the session entitled, “Core Communication: Building Better Science Communication in Cores,” Taylor Edwards and Laura Perry from the University of Arizona and Ioannis Vlachos from Harvard Medical School highlighted practical ways to empower SRR staff to become effective communicators and feel more comfortable in these new roles. In their session “Cells in Motion, Genes in Place,” Steven Polter (University of Rochester), Ken Quayle (Cincinnati Children’s), Brittany Adams (University of Nebraska), and Kaitlyn Petentier (Stowers Institute) highlighted how single cells and spatial genomics are bringing the disciplines of flow cytometry and genomics into collaboration to effectively support and properly validate these complex experiments.

The session entitled “Federal Uncertainty: A one year follow up to the Conversation on SRR Sustainability,” followed up on the extremely well attended session from ABRF 2025, which also led to the JBT Outstanding Manuscript of the Year, “Voices from the Bench: Focus Group Insights on Shared Research Resource Sustainability Amid Federal Policy Shifts.”^1^ This year, the session was led by Roxann Ashworth from Johns Hopkins University with help from a group of table moderators. The format for the session was developed in consultation with the ABRF Core Administrators Network (CAN), involved a Slido-based survey and a series of questions for discussions at each table with a notetaker, and was approved by the IRB at Cincinnati Children’s, the home affiliation of the PI of the overall study (Nicole White). This was another cross-disciplinary session with relevance to staff career development. This session’s balanced structure was focused upon generating results suited for rigorous analysis with the potential for open-ended discussion. All who attended were invited to participate and given space to express their personal experiences and perspectives.

The final keynote was presented by Bryan Welm from the University of Utah, Huntsman Cancer Institute. Professor Welm’s lab has produced seminal work on stem cell dysregulation and breast cancer transformation. However, Professor Welm’s presentation focused upon his amazing work as a sculptor, particularly in making 3D metal representations of biomedically relevant molecules, which he often presents to researchers who have devoted their professional lives to understanding those specific molecules. Dr. Welm’s fantastic presentation reminded us of the importance of visualization and artistic representation when conveying complex scientific ideas and how scientists can broaden the portfolio of tools when communicating our work.

The meeting offered several opportunities to highlight technician and staff contributions to their SRRs, most notably through the revamped Lightning Talk and Poster Sessions. Lightning talk sessions highlighted research, development, and administrative workflows in a compact, informative format. Waters Corporation awarded four of these research talks that best exemplified the advancement work that is done in SRRs. Congratulations to Drew Hankiewicz (Van Andel Institute), Aodong Qiu (University of Pittsburgh), Carole Perrot (Johns Hopkins), and Christopher Nagy (UNC - Chapel Hill) for their work! We are enormously grateful for Waters and their long-standing support for exceptional research in SRRs. The return of scientific poster sessions offered an additional opportunity to highlight the work happening at our institutions, to go in depth on these SRR-driven initiatives, to foster conversation, to discuss rigor and reproducibility, and to create moments for transferring skills and ideas among SRR personnel.

Awards are an important and enjoyable annual series of events at ABRF conferences, and this year did not disappoint! Kevin Eliceiri of the University of Wisconsin–Madison was the inaugural recipient of the Spencer Shorte Legacy Award. Spencer passed away in early 2025 and was a legend in the microscopy and SRR community who had a deep impact on all who knew him. Kevin’s plans for the award involve expanding his mentorship program, supporting a topic that was especially important to Spencer.

John R. Yates from the Scripps Research Institute was the recipient of the 2026 ABRF Award in recognition of his transformative work in the field of proteomics. Mariana De Niz from Northwestern University received the 2026 ABRF DEI Award in acknowledgement of her longstanding commitment to expanding access to scientific technology and information, particularly to underserved communities and across language barriers.

Further highlighting the excellence of Northwestern University, the recipient of the 2026 Alan R. Smith Mentor of the Year Award was Constandina Arvanitis, who is Dr. De Niz’s supervisor at Northwestern. The 2026 Robert A. Welch Outstanding Research Group/Committee Member of the Year Award was given to Andrew Vinard from Tufts University for his tireless work in support of numerous ABRF initiatives and activities. Finally, Simon Goldstein from the La Jolla Institute for Immunology was the recipient of the 2026 ABRF Founder’s Award, which is given to support early career SRR staff with the potential for future ABRF leadership.

Attendees left Pittsburgh and the ABRF meeting reinvigorated by the strength, resilience, and collaborative spirit within our SRR community. Across workshops, sessions, posters, and conversations, attendees formulated new relationships, actionable ideas, renewed energy, and practical strategies to take back to their home institutions. The meeting reinforced ABRF’s commitment to expanding opportunities and supporting members at all stages of their SRR career path through a focus on workforce development initiatives, opportunities for mentorship and cross-disciplinary collaboration, and opportunities to showcase work undertaken in individual SRRs. Most importantly, the meeting served as a reminder that the success of SRRs depends not only on innovation and technology, but also on the relationships built between SRRs and the people who dedicate their time to growing these communities of support, training, and shared purpose. We extend tremendous gratitude to the ABRF leadership, committees, sponsors, volunteers, staff, and countless contributors that made this year’s meeting a meaningful event for the ABRF community.
